# A Role for Uric Acid and the Nalp3 Inflammasome in Antiphospholipid Antibody-Induced IL-1β Production by Human First Trimester Trophoblast

**DOI:** 10.1371/journal.pone.0065237

**Published:** 2013-06-06

**Authors:** Melissa J. Mulla, Jane E. Salmon, Larry W. Chamley, Jan J. Brosens, Crina M. Boeras, Paula B. Kavathas, Vikki M. Abrahams

**Affiliations:** 1 Division of Maternal-Fetal Medicine, Department of Obstetrics, Gynecology & Reproductive Sciences, Yale University School of Medicine, New Haven, Connecticut, United States of America; 2 Hospital for Special Surgery and Weill Cornell Medical College, New York, New York, United States of America; 3 Department of Obstetrics and Gynecology, University of Auckland, Auckland, New Zealand; 4 Division of Reproductive Health, Clinical Sciences Research Institute, University of Warwick, Coventry, United Kingdom; 5 Departments of Laboratory Medicine and Immunobiology, Yale University School of Medicine, New Haven, Connecticut, United States of America; Medical Faculty, Otto-von-Guericke University Magdeburg, Medical Faculty, Germany

## Abstract

Women with antiphospholipid syndrome (APS) are at risk of recurrent pregnancy loss and obstetrical disorders, such as preeclampsia and intrauterine growth restriction (IUGR). Antiphospholipid antibodies (aPL) directly target the placenta by binding beta_2_-glycoprotein I (β_2_GPI) expressed on the trophoblast. We recently demonstrated in human first trimester trophoblast cells that anti-β_2_GPI antibodies (Abs) induce the secretion of IL-1β in a Toll-like receptor 4 (TLR4)-dependent manner. IL-1β secretion requires processing of pro-IL-1β and this is mediated by the inflammasome, a complex of Nalp3, apoptosis-associated speck-like protein containing a CARD (ASC) and caspase-1. The objective of this study was to determine if aPL induce IL-1β production in trophoblast via the inflammasome. Using a human first trimester trophoblast cell line, we demonstrated that a mouse anti-β_2_GPI mAb and human polyclonal aPL-IgG induce IL-1β processing and secretion, which was partially blocked upon caspase-1 inhibition. Nalp3 and ASC knockdown also attenuated anti-β_2_GPI Ab-induced IL-1β secretion. Furthermore, aPL stimulated the production of uric acid in a TLR4-dependent manner; and inhibition of uric acid prevented aPL-induced IL-1β production by the trophoblast. These findings demonstrate that aPL, via TLR4 activation, induce a uric acid response in human trophoblast, which in turn activates the Nalp3/ASC inflammasome leading to IL-1β processing and secretion. This novel mechanism may account for the inflammation at the maternal-fetal interface, which causes placental dysfunction and increases the risk of adverse pregnancy outcome in patients with APS.

## Introduction

Women with antiphospholipid syndrome (APS) and antiphospholipid antibodies (aPL) are at risk for adverse pregnancy outcomes, such as recurrent miscarriage, fetal demise, placental insufficiency, preeclampsia, and intrauterine growth restriction (IUGR) [1]. Even though pathologic aPL are the common underlying cause, systemic APS is characterized as primarily a pro-thrombotic disease, while obstetric APS is primarily a pro-inflammatory syndrome [2], . Studies of aPL-associated pregnancy failure in mice have demonstrated a key role for complement-mediated injury; and for tissue factor-mediated inflammation, triggering neutrophil activation and tumor necrosis factor alpha (TNFα) production [4]–[7]. In humans, however, the precise mechanisms are still not as well understood. Nonetheless, there also appears to be an inflammatory element [8]–[13], with the placental trophoblast potentially playing a central role [14].

Antiphospholipid antibodies (aPL) target the placenta by binding beta_2_-glycoprotein I (β_2_GPI) expressed by the trophoblast, and can directly alter the function of these cells [14]–[17]. We recently demonstrated that anti-β_2_GPI antibodies (Abs) induce secretion of interleukin-1 beta (IL-1β) by human first trimester trophoblast cells through the innate immune receptor, Toll-like receptor 4 (TLR4), and its adaptor protein, MyD88 [16]. IL-1β is a potent pro-inflammatory cytokine which is important for mediating host immune responses towards infection [18]. However, if IL-1β production is excessive or not appropriately controlled, it can lead to tissue damage and pathology [19]. Indeed various autoimmune diseases have been associated with elevated IL-1β [20]. Moreover, elevated placental IL-1β has been associated with pregnancy complications, such as preterm birth and preeclampsia [21]–[23].

Since IL-1β has the potential to be damaging, its regulation is tightly controlled. Unlike most other cytokines, IL-1β production involves a two-step process. The first step requires induction of pro-IL-1β expression. This is thought to be triggered through innate immune signals, such as TLRs. Once expressed, pro-IL-1β can then be cleaved into its active form and secreted [24]. This second step is typically mediated by the inflammasome, a protein complex that facilitates pro-IL-1β processing. The most well characterized is the Nalp3 inflammasome which, in addition to the Nod-like receptor, Nalp3 (Nlrp3), contains apoptosis-associated speck-like protein containing a CARD (ASC) and caspase-1 [25]. Once the inflammasome has assembled, caspase-1 becomes activated and in turn cleaves pro-IL-1β [24]. The Nalp3/ASC inflammasome is expressed by human first trimester trophoblast cells and mediates IL-1β production in response to the Nalp3 agonist and host danger signal, monosodium urate (uric acid) [26]. Therefore, the objective of this study was to understand the molecular basis for aPL-induced IL-1β secretion by the trophoblast, and to determine if the inflammasome is involved.

## Materials and Methods

### Reagents and Antibodies

The caspase -1 inhibitor (Z-WEHD-FMK) was purchased from R&D Systems (Minneapolis, MN). Uricase was purchased from Sigma Aldrich (St Louis, MO). The TLR4 antagonist, LPS from *R. sphaeroides* (LPS-RS) was purchased from Invivogen (San Diego, CA). The rabbit polyclonal antibody to IL-1β (#2022), which recognizes both the full-length pro and the processed active forms, was purchased from Cell Signaling Inc. (Danvers, MA). The rabbit polyclonal antibody for human β-actin was purchased from Sigma.

### Patient Samples

Serums were collected as part of The **PROMISSE** Study (**P**redictors of p**R**egnancy **O**utcome: bio**M**arkers **I**n antiphospholipid antibody **S**yndrome and **S**ystemic lupus **E**rythematosus) [13], [27]. This is an ongoing multicenter, National Institutes of Health-funded prospective observational study of pregnancies of women with aPL, and systemic lupus erythematosus (SLE), or both, as well as healthy pregnant controls. Patients were followed monthly through their pregnancies and data and samples collected. Seven study sites recruited consecutive pregnant women referred because of a suspected diagnosis of aPL and/or SLE in the first trimester of pregnancy. Healthy pregnant controls, selected among women with no known illness, no prior fetal loss, no more than one embryonic loss, and at least one successful pregnancy, were recruited and studied in parallel to the patient groups. This paper concerns a subset of participants who have aPL (n = 55) and healthy pregnant controls (n = 113). The Institutional Review Board at each of the PROMISSE Study sites approved participation of patients. Written informed consent was obtained from all participants.

Inclusion criteria were: confirmed positive aPL at two or more separate time points and at least one positive result in the core laboratories (see below) during pregnancy, live intrauterine pregnancy, confirmed by ultrasound, age 18–45 years, ability to give informed consent, and hematocrit >26%. Exclusion criteria, chosen so that other non-aPL causes of adverse pregnancy outcome would not confound the findings were: treatment with prednisone >20 mg/day, urine protein ≥1000 mg in 24 hours or protein/creatinine ratio ≥1000 mg protein/gram creatinine on spot urine sample; serum creatinine >1.2 mg/dL; type I or II diabetes mellitus antedating pregnancy, blood pressure ≥140/90 mm mercury at the screening visit, and multi-fetal pregnancy. PROMISSE patients with SLE were excluded from this study. Patients were evaluated monthly by an obstetrician and each trimester by a rheumatologist through three months postpartum with physician examination, questionnaires, obstetric ultrasounds and laboratory testing.

In order to be classified as aPL^+^, at least one of the following aPL criteria were present at screening or within 2 visits post-screening as determined by the study core labs: 1) aCL (anti-cardiolipin): IgG ≥40 GPL units; IgM ≥40 MPL units; 2) Positive LAC (lupus anticoagulant): RVVT (Russell viper venom time), Kaolin, dilute TTI (tissue thromboplastin inhibition) or PTT LA (sensitive partial thromboplastin time lupus anticoagulant); 3) Anti-β_2_GPI (anti-beta_2_glycoprotein I): IgG ≥40 GPL units; IgM ≥40 MPL units; and at least one of the following must be present: 1) History of positive aPL pre-pregnancy at local or core lab [History of Moderate/High aCL (IgG >25 GPL units; IgM >25 MPL units); 2) History of positive LAC (positive RVVT, Kaolin, dilute TTI or PTT LA); 3) History of Moderate/High anti-β_2_GPI (IgG >25 GPL units; IgM >25 MPL units)], between six weeks and five years prior to the screening visit; 4) A second positive aPL during pregnancy (meeting the above aPL criteria); 5) A second positive aPL at 3MPP (meeting the above aPL criteria).

Primary study outcomes were defined as the occurrence of one or more of the following: 1) otherwise unexplained fetal death; 2) neonatal death prior to hospital discharge and due to complications of prematurity; 3) indicated preterm delivery prior to 36 weeks’ gestation because of gestational hypertension, preeclampsia or placental insufficiency; 4) birth weight <5^th^ %ile and/or delivery before 36 weeks because of IUGR and confirmed by birth weight <10^th^ %ile. Serums collected from 20–23 weeks gestation were analyzed for uric acid levels. From the aPL^+^ group, 10 of 55 aPL^+^ patients had an adverse pregnancy outcome (3 fetal deaths, 7 preeclampsia, 2 IUGR), while 4 of 113 healthy controls (aPL^−^) had an adverse pregnancy outcome (1 fetal death, 2 preeclampsia, 1 IUGR).

### Trophoblast Cells

The human first trimester trophoblast telomerase-transformed cell line, Sw.71 [28], was used in these studies. Cells were cultured in DMEM (Gibco-Invitrogen; Grand Island, NY), supplemented with 10% fetal bovine serum (Hyclone, South Logan, UT), 10 mM Hepes, 0.1 mM MEM non-essential amino acids, 1 mM sodium pyruvate and 100 nM penicillin/streptomycin (Gibco-Invitrogen). Cells were maintained at 37°C/5% CO_2_. The Sw.71 cells were stably transfected with the pLKO.1 expression plasmids containing the ASC-shRNA construct, NM_013258.3-718s1c1 (sh-ASC), or a non-target shRNA control, SHC002 (sh-control) from Sigma Aldrich (St Louis, MO), as previously described [26], [29]. The Sw.71 cells were also transfected with specific shRNA Nalp3-shRNA (sh-Nalp3) or a mutated targeting sequence as a negative control (sh-mut), as previously described [29].

### Antiphospholipid Antibodies

The mouse IgG1 anti-human β_2_GPI monoclonal antibody (anti-β_2_GPI mAb), IIC5, was used in these studies. This antibody was produced by one of us (LWC), under sterile conditions, and was filter-sterilized prior to use. IIC5 was cloned from mice immunized with purified human β_2_GPI, and have been previously characterized [30]. Like human aPL, IIC5 binds β_2_GPI, but only when it is immobilized on a suitable negatively charged surface, such as the phospholipids, cardiolipin or phosphatidyl serine, or irradiated polystyrene [31]. IIC5 binds to first trimester trophoblast cells, and similarly to patient-derived polyclonal aPL, alter trophoblast function [15], [16]. Mouse IgG1, clone 107.3 (BD Pharmingen, San Jose) served as an isotype control. In addition, patient-derived total IgG containing aPL (aPL-IgG) was used. This aPL-IgG was isolated from the sera of a patient with APS, which was characterized as having high-titer aPL (>140 GPL U), thromboses, and/or pregnancy losses, with confirmed β_2_GPI activity. Normal human IgG negative for aPL served as a control (Sigma).

### Western Blot Analysis

For analysis of proteins by Western blot, samples were diluted with gel loading buffer and boiled for 5 minutes, after which they were resolved under reducing conditions on 12% SDS-PAGE gels and then transferred onto PVDF membrane (PerkinElmer, Boston, MA). Membranes were blocked with 5% fat-free powdered milk (FFPM) in PBS/0.05% Tween-20 (PBS-T). Following washes with PBS-T, membranes were incubated overnight at 4°C with primary antibody in PBS-T/1% FFPM. Following this incubation, membranes were washed as before and then incubated with the goat anti-rabbit IgG secondary antibody conjugated to peroxidase (Vector Labs; Burlingame, CA) in PBS-T/1% FFPM. Following washes with PBS-T and then with distilled water, the peroxidase-conjugated antibody was detected by enhanced chemiluminescence (PerkinElmer). β-actin was used as internal control, in addition to Ponceau Red, to validate the amount of protein loaded onto the gels. Images were recorded and semi-quantitative densitometry performed using the Gel Logic 100 and Kodak MI software (Eastman Kodak, Rochester, NY).

### Measurement of IL-1β, IL-8, and Uric Acid

Trophoblast culture supernatants were analyzed for IL-1β using an ELISA kit from R&D Systems, for IL-8 using an ELISA kit from Enzo Life Sciences (Farmingdale, NY). For the measurement of uric acid, culture supernatants (secreted) and cell lysates (intracellular) were analyzed using the QuantiChrom assay kit from BioAssay Systems (Hayward, CA). Uric acid levels in patient serum were measured by local clinical chemistry laboratories according to standard procedures.

### Statistical Analysis

Data are expressed as mean ± SD unless otherwise stated. Statistical significance (*p*<0.05) was determined using either Student’s *t*-tests, Mann-Whitney *U* test, for multiple comparisons, one-way ANOVA followed by Bonferroni’s post-hoc test using Instat and Prism Graphpad software (La Jolla, CA).

## Results

### Antiphospholipid Antibodies Induce Trophoblast IL-1β Processing and Secretion in a caspase-1 Dependent Manner

**Figure 1:**
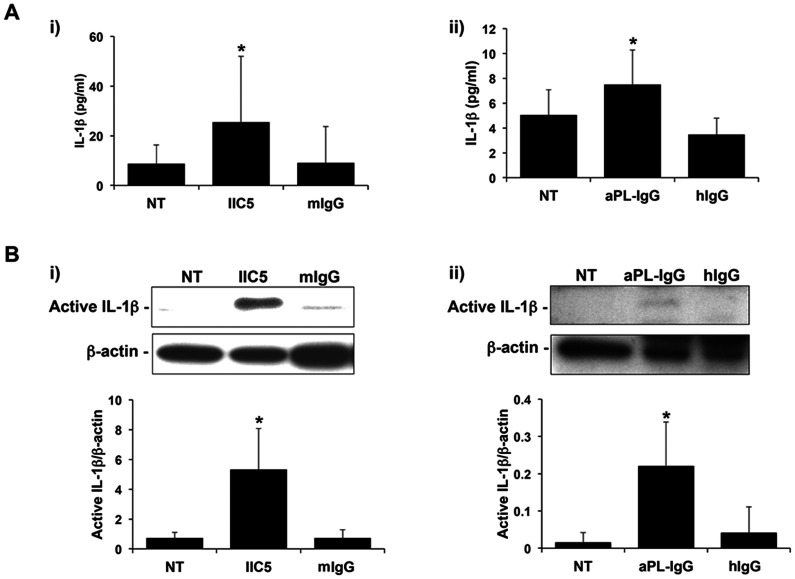
Antiphospholipid antibodies induce trophoblast IL-1β processing and secretion. Trophoblast cells were either not treated (NT) or treated with (i) the anti-β_2_GPI mAb, IIC5 (20 µg/ml) or mouse IgG1 control (mIgG; 20 µg/ml); or (ii) human aPL-IgG (500 µg/ml) or normal human IgG control (hIgG; 500 µg/ml) for 72 hrs. (A) Barcharts show levels of secreted IL-1β as determined by ELISA. Data are from 5 independent experiments. *p<0.01 versus the NT control. (B) Trophoblast cells were evaluated for active IL-1β (17 kDa) expression by Western blot (representative blots are shown). Barcharts below show quantification of protein expression as determined by densitometry and normalized to β-actin. Data are from 3 independent experiments. *p<0.05 versus the NT control.

**Figure 2:**
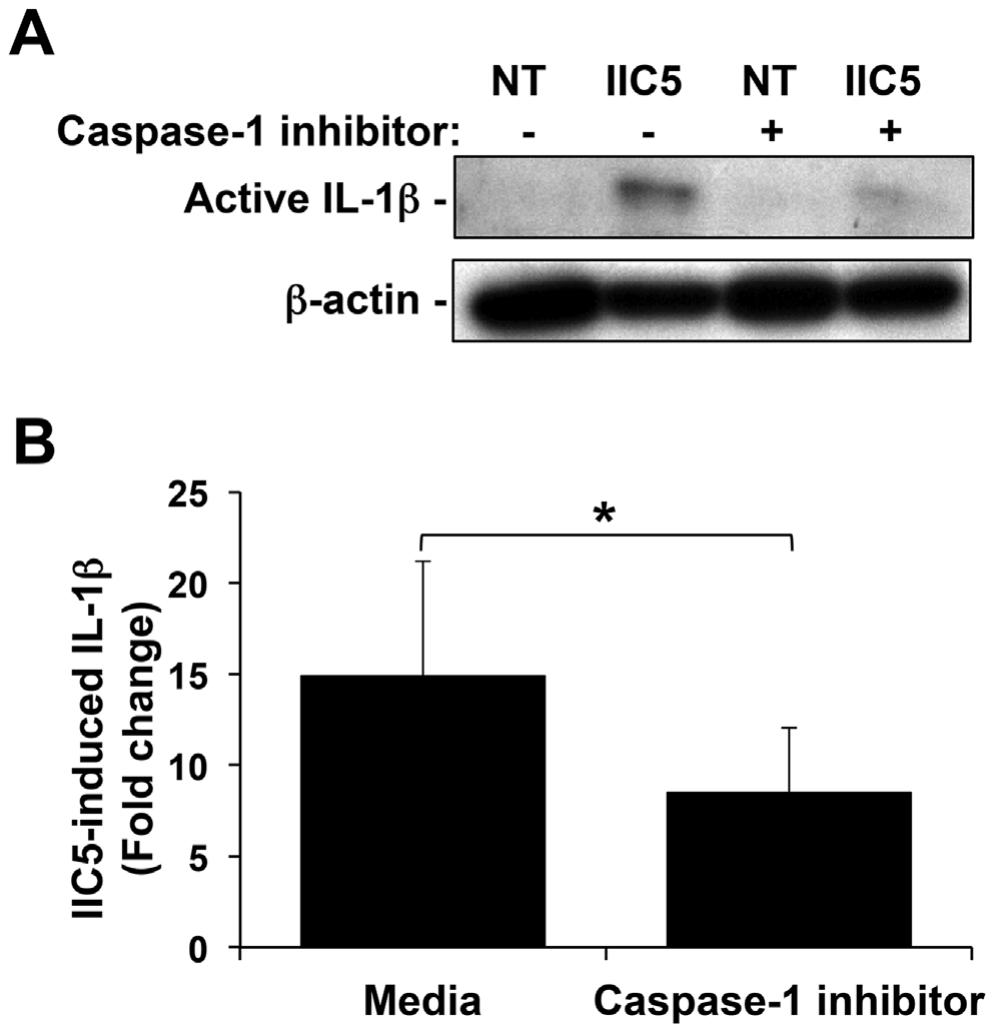
Antiphospholipid antibody-induced trophoblast IL-1β is dependent upon caspase-1. Trophoblast cells were either not treated (NT) or treated with the anti-β_2_GPI mAb, IIC5 (20 µg/ml), all in the presence of media or the caspase-1 inhibitor (5 µM) for 72 hrs. (A) Active IL-1β (17 kDa) expression was evaluated by Western blot. (B) Barchart shows IIC5-induced IL-1β secretion as determined by ELISA and expressed as fold change relative to the untreated control. Data are from 4 independent experiments (*p<0.05).

IL-1β secretion can only occur after pro-IL-1β is processed into its active form. This is commonly mediated by the Nalp3 inflammasome; a complex of Nalp3 and ASC that subsequently activates caspase-1, leading to pro-IL-1β processing [24]. We, therefore, examined whether our previous observation of aPL-mediated IL-1β secretion by the trophoblast [16] was associated with its processing. As shown in Figure 1A, the first trimester trophoblast cell line, Sw.71, secreted significantly elevated levels of IL-1β: (i) in response to the mouse anti-β_2_GPI mAb, IIC5; and (ii) in response to patient-derived aPL-IgG. The mouse and human IgG controls had no effect on trophoblast IL-1β secretion (Figure 1A; i & ii). After treatment with the mouse anti-β_2_GPI mAb, IIC5, but not the mouse IgG control (mIgG), expression of the cleaved active form of IL-1β (17 kDa) significantly increased, as determined by Western Blot analysis (Figure 1B; i). We also observed a decrease in the endogenous expression of the pro-form of IL-1β (31 kDa) after treatment with IIC5, when compared to the NT and mouse IgG controls. However, because of a high level of background this was difficult to quantify (data not shown). Similarly, treatment of trophoblast cells with human aPL-IgG, but not the human IgG control, significantly increased expression of active IL-1β (Figure 1B; ii). Because of high background caused by the aPL-IgG and IgG control preparations, we were unable to clearly see the pro-IL-1β band (data not shown). Having established trophoblast IL-1β processing and secretion in response to aPL, we sought to determine the role of caspase-1. The presence of a caspase-1 inhibitor significantly reduced IIC5-induced IL-1β processing (Figure 2A), and IIC5-induced IL-1β secretion (Figure 2B).

### Antiphospholipid Antibody-induced Trophoblast IL-1β is Dependent on ASC and Nalp3

**Figure 3:**
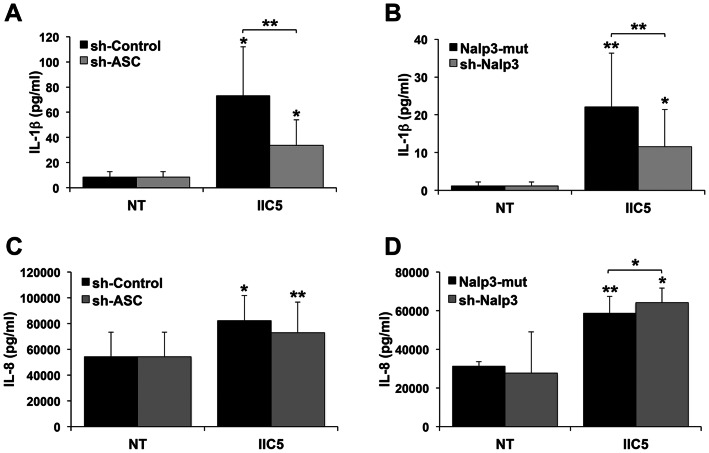
Antiphospholipid antibody-induced trophoblast IL-1β is dependent on ASC and Nalp3. Trophoblast transfected to express either (A & C) shRNA for ASC (sh-ASC) or a control sequence (sh-control) (n = 5); or (B & D) shRNA for Nalp3 (sh-Nalp3) or a Nalp3 mutated targeting sequence (Nalp3-mut) (n = 4), were either not treated (NT) or treated with the anti-β_2_GPI mAb, IIC5 (20 µg/ml) for 72 hrs. Culture supernatants were measured for (A & B) IL-1β and (C & D) IL-8 by ELISA. *p<0.05; **p<0.001 versus the NT control, unless indicated otherwise.

Since aPL-induced IL-1β production by the trophoblast appeared to utilize caspase-1, we next determined the role of the Nalp3/ASC inflammasome in this process. We previously reported that the specific Nalp3 agonist, monosodium urate (uric acid) [25] can trigger IL-1β production via ASC and Nalp3 in the Sw.71 trophoblast cell line, indicating that the Nalp3 inflammasome is functional in these cells [26], [29]. Thus, for this current study, we used the same Sw.71 cell lines expressing either: (A & C) shRNA specific for ASC (sh-ASC) or a non-targeting ASC control (sh-control); or (B & D) shRNA specific for Nalp3 (sh-Nalp3) or a non-targeting mutant control (Nalp3-mut). The sh-ASC and sh-Nalp3 expressing trophoblast cells have been characterized to exhibit reduced ASC and Nalp3 expression, respectively, as well as reduced function [26], [29]. As shown in Figure 3, knockdown of (A) ASC, or (B) Nalp3 significantly reduced IIC5-induced IL-1β secretion. However, knockdown of (C) ASC, or (D) Nalp3 did not reduce IIC5-induced IL-8 secretion (Figure 3).

### Antiphospholipid Antibodies Induce Trophoblast Uric Acid Production via TLR4, which in Turn Triggers IL-1βproduction

**Figure 4:**
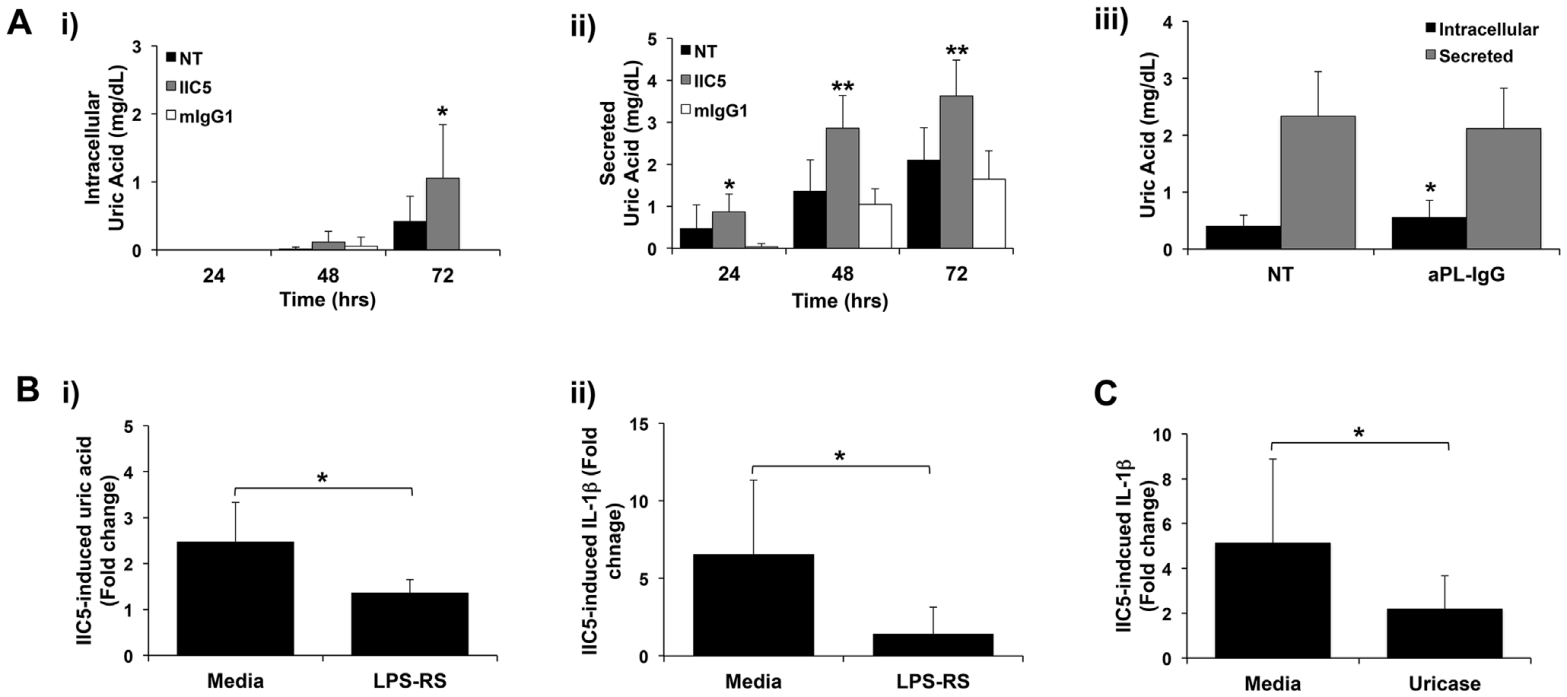
Antiphospholipid antibodies induce trophoblast uric acid production by trophoblasts via TLR4, which in turn triggers IL-1βproduction. (A) Trophoblast cells were either not treated (NT) or treated with (i & ii) the anti-β_2_GPI mAb, IIC5 (20 µg/ml) or mouse IgG1 control (mIgG; 20 µg/ml) for 24, 48 and 72 hrs; or (iii) aPL-IgG (500 µg/ml) for 72 hrs. Cell lysates (intracellular) and supernatants (secreted) were measured for uric acid. Data are from 4–6 independent experiments. *p<0.05; **p<0.001 versus the NT control. (B) Trophoblast cells were pretreated with either media or the TLR4 antagonist, LPS-RS (10 µg/ml) for 30 mins before cells were then treated with or without the anti-β_2_GPI mAb, IIC5 (20 µg/ml) for 72 hrs. Barcharts show levels of IIC5-induced: (i) secreted uric acid and (ii) secreted IL-1β, expressed as fold change relative to the untreated control. Data are from 4 independent experiments (*p<0.01). (C) Trophoblast cells were treated with or without the anti-β_2_GPI mAb, IIC5 (20 µg/ml) in the presence of media or uricase for 72 hrs. Barchart shows levels of IIC5-induced IL-1β secretion, expressed as fold change relative to the untreated control. Data are from 3 independent experiments (*p<0.05).

We next questioned how aPL, binding to the trophoblast [16], are able to trigger activation of the Nalp3 inflammasome. We previously reported that aPL induce IL-1β secretion via TLR4 and MyD88 [16]. We, therefore, tested the hypothesis that through TLR4, aPL could induce a factor that subsequently activates Nalp3. Treatment of trophoblast cells with the anti-β_2_GPI mAb, IIC5, significantly upregulated the intracellular expression and the secretion of uric acid (Figure 4A; i). Human aPL-IgG also significantly increased trophoblast intracellular uric acid expression, but not its secretion (Figure 4A; ii). As shown in Figure 4B, blocking of TLR4 using the antagonist, LPS-RS, significantly inhibited (i) uric acid, and (ii) IL-1β production in response to the anti-β_2_GPI mAb, IIC5. To further confirm that aPL-induced uric acid was indeed mediating the IL-1β response, trophoblast cells were treated with IIC5 in the presence or absence of uricase, which degrades uric acid. Uricase significantly inhibited IIC5-induced IL-1β secretion by the trophoblast (Figure 4C).

### Serum Uric Acid Levels are Elevated in aPL^+^ Patients with Adverse Pregnancy Outcomes

To validate our *in vitro* findings, we analyzed the serum from pregnant women with or without aPL at 20–23 weeks gestation for uric acid and compared the levels in those with and without pregnancy outcomes. As shown in Table 1, aPL^+^ patients who had an adverse pregnancy outcome had significantly higher serum uric acid levels than pregnant aPL^+^ patients who had no adverse pregnancy outcome (*p* = 0.008). Furthermore, among aPL^+^ patients, with uric acid >4.7 mg/dL (>2SD above the mean of aPL^−^ patients), 44% (4 out of 9) had adverse outcomes compared to 13% (6 out of 46) with uric acid <4.7 mg/dL (*p*<0.05). There was no significant difference in the serum uric acid levels in healthy controls (aPL^−^) who had an adverse pregnancy outcome when compared with those with normal pregnancies (Table 1), but the number with outcomes was small, thus limiting the power of our analysis. There were no significant differences in the levels of uric acid in sera collected at the time of initial screening in the first trimester (data not shown).

**Table 1:** Uric acid levels in serum from aPL^−^ and aPL^+^ PROMISSE patients at 20–23 weeks gestation.

aPL^−^	No adverse pregnancy outcome	Adverse pregnancy outcome
Number of patients	109	4
Mean uric acid (mg/dL)	3.26	3.95
SEM	0.07	0.32
Range	2.0–6.0	3.1–4.6
*p* value vs. No outcome*		0.053
**aPL^+^**	**No adverse pregnancy outcome**	**Adverse pregnancy outcome**
Number of patients	45	10
Mean uric acid (mg/dL)	3.56	4.54
SEM	0.16	0.33
Range	1.4–7.3	3.1–5.9
*p* value vs. No outcome*		0.008

* Based on Mann-Whitney *U* test.

## Discussion

APS patients are at high risk for recurrent arterial and venous thrombosis [2]. Additionally, women with APS are at risk for recurrent pregnancy loss and late gestational pregnancy complications, like preeclampsia and IUGR [1]–[3]. Since pathologic aPL are strongly associated with thrombosis, historically, pregnancy failure in APS patients was thought to arise from a thrombotic event at the maternal-fetal interface. However, intravascular or intervillous blood clots are not commonly found in miscarriage samples from APS patients [32]. Instead, in humans, there is often insufficient placentation [32], [33], and indications of inflammation at the maternal-fetal interface [8]–[12]. A role for inflammation in the pathogenesis of adverse pregnancy outcome in APS patients has been supported by animal models, with complement activation implicated as a key player in aPL-induced pathology [4]–[7]. *In vitro* studies by our group have further supported a role for inflammation in aPL-associated pregnancy complications by showing that anti-β_2_GPI Abs directly induce human first trimester trophoblast cells to generate an inflammatory cytokine/chemokine response via TLR4/MyD88 [26]. One of the factors induced in the trophoblast by aPL is the potent pro-inflammatory, and potentially damaging cytokine, IL-1β. The objective of our current study was to investigate the molecular mechanism by which aPL induce IL-1β secretion by the trophoblast. Herein, we have demonstrated for the first time that aPL, through TLR4, induces a uric acid response, which in turn activates the Nalp3/ASC inflammasome in the trophoblast, leading to IL-1β processing and secretion.

Using a human first trimester trophoblast cell line (Sw.71), we found that, in addition to inducing IL-1β secretion as previously reported [16], a mouse anti-β_2_GPI mAb and a patient-derived aPL-IgG, triggered IL-1β processing, evidenced by the appearance of the cleaved 17 kDa active form. Although typically, the first step for IL-1β production is the induction of the 31 kDa pro-form, we have previously reported that untreated first trimester trophoblast cells express high levels of pro-IL-1β and, therefore, its induction in these cells may not be a pre-requisite for IL-1β processing and secretion [26]. Indeed, treatment of trophoblast cells with anti-β_2_GPI Ab did not induce pro-IL-1β expression, but instead decreased levels of the endogenous protein, similarly to what we have observed when the cells are treated with the Nalp3 agonist, monosodium urate [26].Thus, the dependency of TLR4 in mediating IL-1β secretion by the trophoblast [16], appears not to be for the induction of pro-IL-1β, as reported in other systems [34].

Using an inhibitor of caspase-1 activity, we demonstrated that aPL-induced IL-1β processing and secretion is regulated by caspase-1, indicating a potential role for the inflammasome in aPL-mediated IL-1β production. Since we previously reported that the Nalp3/ASC inflammasome was expressed and functional in the trophoblast, we tested its role in aPL-induced IL-1β. Anti-β_2_GPI mAb-induced IL-1β secretion, but not IL-8, was reduced when ASC and Nalp3 expression was inhibited. Together these findings indicate a role for the Nalp3/ASC inflammasome in aPL-induced IL-1β production by the trophoblast. However, these findings still did not explain the link between aPL-induced TLR4 activation [16], and the induction of inflammasome activation. We, therefore, postulated that a secondary factor might be produced in response to aPL, under the regulation of TLR4, that could specifically activate Nalp3.

Based on previous findings that exogenous uric acid triggers Nalp3 activation in the trophoblast [26], and that endogenously produced uric acid could activate the Nalp3 inflammasome and IL-1β in other systems [35]–[37], we pursued this as a candidate. Indeed, the anti-β_2_GPI mAb increased intracellular and secreted uric acid levels. Moreover, inhibition of TLR4 using the antagonist LPS-RS, which primarily blocks TLR4/MD2 signaling [38], prevented the anti-β_2_GPI mAb-induced uric acid response, as well as IL-1βsecretion. Patient aPL-IgG also increased intracellular uric acid levels in the trophoblast, however, its secretion was not affected. Why the polyclonal aPL-IgG had no effect on secreted uric acid might be a reflection of their fine specificity and heterogeneity. Alternatively, this might be a question of antibody concentration. Since the patient-derived aPL used in this study are a mixture of aPL-IgG and non-aPL-IgG the concentration of anti-β_2_GPI antibodies in this preparation may be much lower than our monoclonal antibody. Indeed this is reflected in the levels of IL-1β produced; the aPL-IgG generates a less robust response than the mAb. However, what this data does indicate is that both intracellularly produced as well as secreted uric acid might play a role in subsequent inflammasome activation. While we believe this is the first direct demonstration that TLR4 activation triggers uric acid production, a study using monocytic THP-1 cells reported that viral ssRNA, the agonist for human TLR8, induces uric acid [39]. Having determined that aPL induce trophoblast uric acid production via TLR4, we then went on to confirm its role in aPL-mediated IL-1β production. To do this, we tested the effect of uricase, a compound which rapidly degrades uric acid. The presence of uricase inhibited aPL-induced IL-1β secretion. Together, these findings suggest that aPL via TLR4 upregulates intracellular uric acid, which in turn activates Nalp3, leading to inflammasome activation, and thus, IL-1β processing and secretion.

Since the aPL-induction of first trimester trophoblast uric acid appears to play an upstream role in the cell’s IL-1β response, we sought to validate our *in vitro* findings by evaluating serum uric acid levels in aPL^−^ and aPL^+^ patients enrolled in the PROMISSE study [13]. We found that while uric acid levels were similar in the first trimester, aPL^+^ patients who presented with an adverse pregnancy outcome had significantly higher circulating uric acid than those with normal pregnancies at 20–23 weeks gestation. Of aPL^+^ patients with uric acid levels>2 SD above the mean for aPL^−^ patients, 44% had adverse pregnancy outcomes. Although we cannot determine whether elevated uric acid in aPL^+^ patients with pregnancy complications is a cause or consequence of placental dysfunction from our data, taken together they support the notion that the source as well as the target of excess uric acid in aPL-associated adverse pregnancies may be the placenta [40]. That circulating levels of uric acid were not significantly elevated until the second trimester, may be because serum levels of uric acid depend upon its production and clearance [41]. While aPL may be acting on the trophoblast to induce uric acid locally during the first trimester, as well as the second trimester, differences may not be reflected in the circulation until the second trimester because by this point is gestation, filtration may not keep up with the increased production induced by aPL and levels accumulate over time.

In summary, we have shown for the first time that trophoblast IL-1β production in response to aPL involves TLR4 mediated uric acid production, and subsequent Nalp3/ASC inflammasome activity. This may provide a novel mechanism for the induction of inflammation at the maternal-fetal interface leading to placental dysfunction and adverse pregnancy outcome in patients with APS.
